# A probability of benefit (AUC) approach to phase II cancer trials

**DOI:** 10.1186/1745-6215-16-S2-P227

**Published:** 2015-11-16

**Authors:** Paul Silcocks

**Affiliations:** 1 University of Liverpool, Liverpool, UK

## 

For modern cytostatic but non-cytotoxic agents that result in stable disease without tumour shrinkage, traditional RECIST-defined measures of tumour response used in phase II trials may be less suitable than quantitative measures such as Ki67 proliferation index, change in tumour mass, or fluorothymidine (FLT) uptake. Dichotomising such measures is statistically inefficient. A better approach is to use the Mann-Whitney test. For this, assumptions are few, the sample size formula is simple, and it has an intuitive interpretation in terms of Probability of Benefit (ie the chance of a superior outcome on the new intervention). For phase II trials, discrimination between promising and unpromising treatments is more relevant than assessment of definitive outcomes and the common measure of discrimination offered by a test variable (the area under the receiver operating characteristic curve - AUC) is another interpretation of the Mann-Whitney statistic. Standard values of the AUC from diagnostic testing offer a ready-made calibration, and approximate but useful relationships to other common measures exist, including the hazard ratio, Cohen's standardised difference, the odds ratio, Pearson's correlation, and Number needed to Treat. Values of these measures corresponding to AUCs considered useful for diagnosis show that effects need to be much larger than typically used. This may not be a bad thing: sample sizes are smaller, yet if combined with a sensible likelihood ratio criterion for “strong” evidence, the risk of a misleading positive signal is small and the chance of failure in phase III is reduced.

